# Draft Genome Sequence of Plant-Growth-Promoting Rhizobacterium *Serratia fonticola* Strain AU-AP2C, Isolated from the Pea Rhizosphere

**DOI:** 10.1128/genomeA.01022-13

**Published:** 2013-12-05

**Authors:** Usha Devi, Indu Khatri, Navinder Kumar, Deepak Sharma, Srikrishna Subramanian, Adesh K. Saini

**Affiliations:** Shoolini University of Biotechnology and Management Sciences, Department of Biotechnology, Bajhol, Solan, HP, Indiaa; CSIR-Institute of Microbial Technology, Sector 39A, Chandigarh, Indiab

## Abstract

Plant health can be augmented by plant-growth-promoting rhizobacteria (PGPR) that confer biofertilizer, phytostimulation, and biocontrol activities. Herein, we provide the high-quality draft genome sequence of *Serratia fonticola* strain AU-AP2C, a Gram-negative motile PGPR of the pea plant, conferring phosphate solubilization, ammonia production, and antifungal activity against *Fusarium* sp. The 4.9-Mb genome contains genes related to plant growth promotion and synthesis of siderophores.

## GENOME ANNOUNCEMENT

Plant-growth-promoting rhizobacteria (PGPR) are derived from and exert their plant-growth-promoting effects on the roots. PGPR usually must colonize the root surface efficiently and augment plant health via biofertilization, stimulation of root growth, rhizoremediation, and plant stress control and by reducing plant diseases (1). Nonpathogenic PGPR members of the *Serratia* genus have been shown to confer phosphate solubilization (2), indole-3-acetic-acid production, and phytoremediation (3) and could protect plants from flood-induced damage (4). They also exhibit biocontrol activities against bacterial, fungal, and nematodal diseases (3, 5–8). *Serratia fonticola* AU-AP2C, a Gram-negative motile rod, was isolated from the rhizosphere of pea roots and confers traits similar to PGPR (U. Devi, I. Khatri, N. Kumar, L. Kumar, D. Sharma, S. Subramanian, and A. K. Saini, unpublished results). AU-AP2C expresses phosphate solubilization and ammonia production, which are helpful in providing free phosphate and nitrogen to the plants, respectively. AU-AP2C also produces hydrogen cyanide (HCN) and siderophores that are related to the biocontrol activities of PGPR (Devi, Khatri, Kumar, Kumar, Sharma, Subramanian, and Saini, unpublished). Supporting this, we found that AU-AP2C confers antifungal activities against *Fusarium* sp. (Devi, Khatri, Kumar, Kumar, Sharma, Subramanian, and Saini, unpublished), a fungal pathogen of pea plant.

The genome of *S. fonticola* AU-AP2C was sequenced using the Illumina-HiSeq 1000 technology. Sequencing resulted in 26,030,930 paired-end reads (insert size of 350 bp) of length 101 bp. A total of 25,811,601 high-quality reads with approximately 520× coverage were assembled with CLCbio wb6 (word size 45 and bubble size 60) to obtain 47 contigs (*N*_50_, 328,473 bp). The genome-finishing module of CLCbio was used, followed by SSPACE v2.0 scaffolder (9) and GapFiller v1-10 (10). The gap-filled scaffolds thus obtained were broken at the gaps to obtain 34 contigs (*N*_50_, 340,729 bp) of 4,999,819 bp and average G+C content of 54%. The functional annotation was carried out by RAST (Rapid Annotation using Subsystem Technology) (11), tRNA was predicted by tRNAscan-SE 1.23 (12), and rRNA genes by RNAmmer 1.2 (13). The genome contains 3 rRNA genes (5S-23S-16S) and 73 aminoacyl-tRNA synthetase genes. A total of 4,465 coding regions (1,974 genes transcribed from the positive strand and 2,491 from the negative strand) were found in the genome, of which 3,666 (82%) were functionally annotated. The genome coding density is 86% with an average gene length of 928 bp. The annotated genome sequence has 58 genes responsible for motility and chemotaxis, including 15 genes for flagellar motility. Forty-three genes are responsible for phosphorus metabolism. Twenty-one genes are osmotic stress responsive genes, including 4 for osmoregulation and 59 for oxidative stress, to make a total of 131 genes responsible for stress response in this organism.

The functional comparison of the genome sequences available on the RAST server revealed the closest neighbors of *S. fonticola* AU-AP2C to be *S. proteamaculans* 568 (score 542), followed by *S. odorifera* 4Rx13 (score 541), *S. odorifera* DSM 4582 (score 534), and *S. marcescens* Db11 (score 518).

### Nucleotide sequence accession numbers.

This whole-genome shotgun project has been deposited at DDBJ/EMBL/GenBank under the accession number ASZA00000000. The version described in this paper is the first version, ASZA01000000.
